# Exploring mHealth potential to improve kidney function: secondary analysis of a randomized trial of diabetes self-care in diverse adults

**DOI:** 10.1186/s12882-022-02885-6

**Published:** 2022-08-10

**Authors:** McKenzie K. Roddy, Lindsay S. Mayberry, Devika Nair, Kerri L. Cavanaugh

**Affiliations:** 1 Quality Scholars, VA Tennessee Valley Healthcare System, Nashville, TN USA; 2 Division of General Internal Medicine, Department of Medicine, Vanderbilt University Medical Center, Nashville, TN USA; 3 Division of Nephrology, Department of Medicine, Vanderbilt University Medical Center, Nashville, TN USA

**Keywords:** Type 2 diabetes (T2D), Chronic kidney disease (CKD), mHealth

## Abstract

**Background:**

Many individuals living with chronic kidney disease (CKD) have comorbid Type 2 diabetes (T2D). We sought to explore if efficacious interventions that improve glycemic control may also have potential to reduce CKD progression.

**Methods:**

REACH is a text message-delivered self-management support intervention, which focused on medication adherence, diet, and exercise that significantly improved glycemic control in *N* = 506 patients with T2D. Using data from the trial, we characterized kidney health in the full sample and explored the intervention’s effect on change in estimated glomerular filtration rate (eGFR) at 12 months in a subsample of N=271 patients with eGFR data.

**Results:**

In a diverse sample with respect to race/ethnicity and socioeconomic status, 37.2% had presence of mild or heavy proteinuria and/or an eGFR < 60 mL/min/1.73 m^2^. There was a trending interaction effect between intervention and presence of proteinuria at baseline (b = 6.016, *p* = .099) such that patients with proteinuria at baseline who received REACH had less worsening of eGFR.

**Conclusions:**

Future research should examine whether diabetes directed self-management support reduces CKD progression in ethnically diverse individuals with albuminuria. In highly comorbid populations, such as T2D and CKD, text-based support can be further tailored according to individuals’ multimorbid disease self-management needs and is readily scalable for individuals with limited resources.

**Trial registration:**

This study was registered with ClinicalTrials.gov (NCT02409329).

## Background

There is significant comorbidity between type 2 diabetes (T2D) and chronic kidney disease (CKD). By 2040, over 10% of the global population is expected to have diabetes [1, 2], and T2D accounts for 87 to 91% of the global cases [3]. In 2016, the estimated prevalence for T2D was 8.6% of adults in the United States [4], and is expected to increase over the next 20 years [5]. In the United States, diabetes is the leading cause of kidney disease [6], and an estimated 30-40% of individuals with diabetes also have CKD [7, 8]. Increased prevalence of T2D will result in an increased prevalence of CKD, the combination of which results in a significant economic burden [9].

Given these considerations, innovation is required at the crossroads of T2D and CKD [10]. Specifically, there have been calls for better patient screening [11, 12], especially given the lack of awareness of kidney problems among individuals with T2D [13], Additionally, there is need for increased provider awareness of CKD in T2D, which disproportionately affects minorities and individuals with lower education attainment and socioeconomic status (SES) [14]. The 2020 Kidney Disease Improving Global Outcomes (KDIGO) guidelines for the management of diabetes in chronic kidney disease define chronic kidney disease as abnormalities in the structure or function of kidneys with implications for health present for at least 3 months [15]. Further, the 2021 recommendations from the American Diabetes Association include albumin and estimated glomerular filtration rate (eGFR) monitoring annually and, for patients with urinary albumin > 300 mg/g creatinine and/or eGFR 30-60 mL/min/1.73m^2^, monitoring twice annually [16].

Interventions for T2D targeting healthy lifestyle behaviors such as healthy diet, exercise, and medication adherence could also have a protective effect on kidney health. In fact, KDIGO guidelines for management of diabetes in chronic kidney disease, the first on the topic, highlights the need to monitor hemoglobin A1c (HbA1c) and engage in physical activity [15]. These practice guidelines follow reviews that have highlighted the negative effects of low physical activity, obesity, and smoking on health for individuals with T2D [17].

Efforts to capitalize on the promising areas of overlap to positively impact T2D and kidney health have not demonstrated impact on important health outcomes. A systematic review of self-management support interventions for individuals with diabetes (inclusive of Type 1 and 2) and CKD found low quality evidence for their effectiveness improving HbA1c and systolic blood pressure and no evidence for their effectiveness improving diastolic blood pressure, eGFR, or health related quality of life [18]. These findings should be contextualized by methodological challenges such as blinding investigators and participants as well as the small numbers of study per outcome. Specifically, only 8 studies were included in this review over a period of 23 years, highlighting the gap of knowledge and effective interventions for this comorbid population.

There are a number of promising new self-management interventions for T2D which target glycemic control and therefore may also have the potential to slow CKD progression. These interventions present the opportunity to be on the front end of decreasing or eliminating CKD. The Rapid Education/Encouragement and Communications for Health (REACH) intervention is a text message-delivered self-management support intervention developed for and with diverse individuals with T2D receiving care at Federally Qualified Health Centers [19]. Response rates to text messages were high among white (87%) and Black (80%) participants [20]. In a sample of individuals recruited from safety net clinics and primary care clinics from a large academic medical center, REACH, compared to control, reduced HbA1c at 6 months as well as improved medication adherence and diet through 12 months [21]. It is possible that interventions such as REACH that improve glycemic control have undiscovered protective effects on kidney function in an at need population [22]. We aimed to characterize the sample recruited for T2D for REACH regarding kidney function and examine the intervention’s benefits on CKD progression, beyond its established benefits related to lowered HbA1c and improved diabetes self-management.

### Current study

The current study reports secondary analyses from the REACH randomized control trial (RCT; ClinicalTrials.gov NCT02409329). Using the full sample, in our first aim we describe medical comorbidities including kidney function in a sample of patients with established T2D. Second, in a posthoc subset of the trial sample for whom lab values were available in the electronic health record (EHR), our second aim sought to understand the effect of the intervention on eGFR from baseline to 12-months follow-up. We examined the moderating effect of albuminuria to understand intervention effects for those with a higher vs. lower risk for CKD progression. We preliminarily hypothesize that REACH will have a protective effect on eGFR, especially among those with higher risk for CKD progression.

## Methods

### Subjects

There were 506 individuals randomized in the parent trial used here to describe medical comorbidities. As described elsewhere, participants were recruited from adult primary care clinics at Vanderbilt University Medical Center as well as surrounding community clinics from May 2016 to December 2017 to participate in a trial of a text-message delivered diabetes medication adherence intervention [19, 23]. To be eligible participants had to be > = 18 years old, have a cell phone, be able to speak and read English, be prescribed at least one daily diabetes mediation, and be responsible for taking their own medications. Individuals who had a most recent HbA1c value < 6.8% in the electronic health record during the preceding year, failed a brief cognitive screener, were unable to respond to a text message, or had other auditory or communication limitations that would inhibit participation in phone calls were excluded. Informed consent was obtained from all participants. To examine the effect of the intervention on change in eGFR, we included individuals with complete data in the EHR (*N* = 266) in the subset sample; a full explanation of processes is below.

### Procedures

All participants completed self-report measures and an HbA1c test at baseline and at 12 months, the end of the intervention. Medical chart reviews were completed by trained study staff at baseline and end of the intervention to collect lab values when available. Participants were randomized evenly to intervention or control using multivariate matching which balanced variables of interest between conditions including gender, race/ethnicity, SES, insulin use, and baseline HbA1c and diabetes medication adherence [19].

#### Intervention

Participants assigned to the REACH intervention received daily automated text messages for 12 months to their own phones regarding diabetes medication adherence as well as tips for healthy lifestyle including diet and exercise [19]. Messages were tailored to participants’ prescribed medications and targeted patient-identified barriers to medication adherence [24]. Participants received daily interactive messages with weekly tailored feedback on medication adherence based on their responses.

#### Control

Participants assigned to the control condition completed regular assessments including access to HbA1c results, were sent quarterly newsletters, and had access to a REACH study hotline.

### Materials

#### Demographics

Participants self-reported age, gender, race/ethnicity, income, insurance status, years of education, and diabetes duration at baseline.

#### Chart review

Using a standardized list of keywords and International Classification of Diseases (ICD) codes, trained study staff reviewed patients’ medical records at baseline to identify specific medical comorbidities. If keyword or ICD code was present, staff indicated presence of comorbidity in patient’s record. An internist and director of a division of general internal medicine of a large academic medical center was available to adjudicate records as needed.

#### Glycemic control

Participants completed a test of HbA1c at baseline via venipuncture or point-of-care at their clinic or using a HbA1c kit from CoreMedica Laboratories (Lee’s Summit, MO), which are validated against venipuncture [25].

#### Lab values

Study staff collected lab values from participants’ medical records at baseline and follow-up. Values for microalbumin tests, microalbumin creatinine ratios (ACR), and dipstick urinalyses were recorded depending on what was available within the medical record. Most measures of albuminuria were collected via ARC (72%) with fewer collected via microalbumin (9.7%) and dipstick (18.3%). Serum creatinine values within the last year were recorded at baseline and follow-up.

### Data handling

To be eligible for the Aim 2 analyses, via chart review, participants must have had lab values of albuminuria at baseline in order to define evidence of kidney disease, serum creatinine at baseline and follow-up to assess change in eGFR, baseline eGFR < 15 mL/min/1.73 m^2^ to exclude individuals with end stage renal disease, and time between baseline and follow up eGFR values > 90 days and < 540 days to permit time for potential effect during the study observation period. Of the 506 individuals represented in the larger RCT, 271 individuals were eligible for the subset analysis. Reasons for exclusion were missing baseline albuminuria (*N* = 101), missing baseline or follow-up eGFR (*N* = 98), lab values outside time window (pre to post < 90 or > 540 days; *N* = 33), and baseline eGFR < 15 mL/min/1.73 m^2^ (*N* = 2). One individual was excluded for a decline in eGFR > 100 mL/min/1.73m^2^ at 12-months and was considered an inaccurate outlier. The final sub-study sample size was 271 individuals, who were roughly equally distributed between intervention (*N* = 132) and control (*N* = 139). On average, baseline and follow-up eGFR were collected 326 days apart (SD = 97.05, Interquartile range [264, 391]).

#### Albuminuria

Lab values for albuminuria were dichotomized to create two groups of participants: normal/no albuminuria and any/high albuminuria. Specifically, the no albuminuria group had negative dipstick, albumin–creatinine ratio (ACR) < 30 mg/g, or microalbumin < 30 mg/dL [26, 27]. The any/high albuminuria group had positive (1+ or 2+) dipstick, > = 30 mg/g ACR, or microalbumin > = 30 mg/dL.

#### Serum creatinine

An estimated glomerular filtration rate (eGFR) was calculated using CKD refit equation [28, 29], from serum creatinine, age, and gender.

#### Analytic method

Descriptive statistics were used to report participants’ baseline eGFR and degree of albuminuria. Albuminuria was missing for 47% of the sample at follow-up and therefore is not reported. Standardized mean differences were used to compare the sub-study sample to excluded individuals from the full sample. Linear regression with heteroscedasticity adjusted standard errors was used to predict follow-up eGFR, adjusting for baseline eGFR. The outcome was follow-up eGFR predicted by baseline eGFR, intervention, baseline categorical albuminuria (none versus any), and an interaction between intervention and baseline categorical albuminuria to test for differential effects of the intervention on individuals with varying risks for CKD progression. To follow-up the results of the regression, non-parametric tests of differences (Mann-Whitney U-tests) were used to compare REACH to control separately for individuals with and without albuminuria at baseline on change in eGFR. The parent trial was powered to detect a 0.5% difference in HbA1c between REACH and control [19], and not planned to address this exploratory analysis.

## Results

### Aim 1: kidney function in T2D

The 506 individuals from the parent trial were in their mid-fifties (M = 55.94, SD = 9.57), about half male (45.8%), and most were non-white including 39.1% Black and 6.1% Hispanic. The majority of the sample (77.3%) had an income <$55,000 per year and about half had private insurance (51.2%). On average, participants reported 14 years of school and having had diabetes for about 11 years (Table 1). Per review of the medical chart, hypertension was listed for 78.3% of the full sample, dyslipidemia noted for 60.5, 17.6% had neuropathy, 14.4% had cardiovascular problems, and 1.6% had an amputation, all below the knee.

**Table 1:** Description of participants

Variable	Full Sample M (SD) or N (%)	Sub-study Sample M (SD) or N (%)
Age (years)	55.94 (9.57)	57.31 (8.87)
Male	232 (45.8%)	122 (45.0%)
Race/Ethnicity		
Non-Hispanic white	242 (47.8%)	133 (49.1%)
Black	198 (39.1%)	104 (38.4%)
Hispanic	31 (6.1%)	14 (5.2%)
Other	32 (6.3%)	18 (6.6%)
Non-Hispanic, missing race	3 (0.6%)	2 (0.7%)
Income		
> $55,000	115 (22.7%)	68 (25.1%)
$25,000-$54,499	126 (24.9%)	70 (25.8%)
$10,000-$24,999	131 (25.9%)	69 (25.5%)
< $9999	92 (18.2%)	45 (16.6%)
Missing	42 (8.3%)	19 (7.0%)
Years of School	14.06 (3.15) ^a^	14.27 (3.11)^e^
Insurance Status		
Private Insurance	259 (51.2%)	149 (55.0%)
Public Insurance	126 (24.9%)	67 (24.7%)
Insured – Type Unknown	4 (0.8%)	3 (1.1%)
Uninsured	117 (23.1%)	52 (19.2%)
Diabetes Duration (years)	11.04 (7.93) ^b^	11.45 (7.72)^f^
Baseline HbA1c (%)	8.58 (1.81)^c^	8.51 (1.75)^e^
Baseline eGFR	79.64 (23.36)^d^	77.56 (22.41)
Categorical Baseline eGFR		
Stage 1 (> 90 mL/min)	183 (36.2%)	93 (34.3%)
Stage 2 (60-89.9 mL/min)	191 (37.7%)	113 (41.7%)
Stage 3A (45-59.9 mL/min)	60 (11.9%)	42 (15.5%)
Stage 3B (30-44.9 mL/min)	29 (5.7%)	18 (6.6%)
Stage 4 (15-29.9 mL/min)	5 (1.0%)	5 (1.8%)
Missing	38 (7.5%)	n/a
Baseline Albuminuria		
Normal (negative dipstick, ACR < 30 mg/g, or microalbumin < 30 mg/dL)	261 (51.6%)	197 (72.7%)
Mild (1+ dipstick, 30-300 mg/g ACR, or microalbumin > = 30 mg/dL)	81 (16.0%)	57 (21.0%)
Heavy (2+ dipstick, > 300 mg/g ACR)	26 (5.1%)	17 (6.3%)
Missing	138 (27.3%)	n/a

Sample sizes are 506 for the full sample and 266 for the sub-study sample except where otherwise noted: ^a^N = 498, ^b^N = 499, ^c^N = 495, ^d^N = 472, ^e^N = 266, ^f^N = 267. ^e^GFR = estimated Glomerular filtration rate. ACR = albumin-creatinine ratio. Percent missing for categorical baseline eGFR and baseline albuminuria are not applicable (n/a) for the sub-study sample as values were required for inclusion

At baseline, 51.6% of the full sample had normal levels of albuminuria (i.e. no albuminuria; negative dipstick, albumin-creatinine ratio (ACR) < 30 mg/g, or microalbumin < 30 mg/dL) while 16.0% had mild albuminuria [positive (1+) dipstick, 30-300 mg/g ACR, or microalbumin > = 30 mg/dL], and 5.1% had heavy albuminuria [positive (2+) dipstick, > 300 mg/g ACR; see Table 1]. The average eGFR was 76.20 mL/min/1.73 m^2^ (SD = 23.03). Most of the full sample (69.1%) had an eGFR > 60 mL/min/1.73 m^2^ (Table 1). At baseline, 33.6% had presence of mild or heavy albuminuria and/or eGFR < 60 mL/min/1.73 m^2^, indicating possible CKD, though earlier values were unavailable to confirm CKD. However, presence of kidney disease was documented in the medical record for only 8.1% of the sample via review of ICD codes.

### Aim 2: T2D intervention effect on eGFR

We first compared individuals who met the inclusion criteria for the sub-study analyses to individuals who were excluded from the sub-study analyses on all demographic indicators and baseline lab values. There were no differences on gender, race/ethnicity, income, years of school, insurance status, diabetes duration, glycemic control, or baseline albuminuria between the sub-study sample and excluded individuals from the full sample (all standardized mean differences < .20). There were differences such that younger individuals (SMD = .31), individuals with higher baseline eGFR (SMD = .21), and uninsured individuals (SMD = .20) were more frequently excluded. See Table 2 for baseline variables for the intervention and control groups in the subsample.

**Table 2:** Description of subsample participants

Variable	Intervention M (SD) or N (%)	Control M (SD) or N (%)
Age (years)	57.23 (9.24)	57.38 (8.53)
Male	57 (43.2%)	65 (46.8%)
Race/Ethnicity		
Non-Hispanic white	66 (50.0%)	67 (48.2%)
Black	49 (37.1%)	55 (39.6%)
Hispanic	8 (6.1%)	5 (3.6%)
Other	9 (6.8%)	10 (7.2%)
Non-Hispanic, missing race	0 (0.0%)	2 (1.4%)
Income		
> $55,000	35 (26.5%)	33 (23.7%)
$25,000-$54,499	34 (25.8%)	36 (25.9%)
$10,000-$24,999	33 (25.0%)	36 (25.9%)
< $9999	20 (15.2%)	25 (18.0%)
Missing	10 (7.6%)	9 (6.5%)
Years of School	14.15 (2.74)	14.38 (3.42)
Insurance Status		
Private Insurance	73 (55.3%)	76 (54.7%)
Public Insurance	34 (25.8%)	33 (23.7%)
Insured – Type Unknown	2 (1.5%)	1 (0.7%)
Uninsured	23 (17.4%)	29 (20.9%)
Diabetes Duration (years)	11.65 (7.73)	11.26 (7.73)
Baseline HbA1c (%)	8.46 (1.68)	8.55 (1.82)
Baseline eGFR	77.12 (22.49)	77.99 (22.41)
Categorical Baseline eGFR		
Stage 1 (> 90 mL/min)	43 (32.6%)	50 (36.0%)
Stage 2 (60-89.9 mL/min)	60 (45.5%)	53 (38.1%)
Stage 3A (45-59.9 mL/min)	15 (11.4%)	27 (19.4%)
Stage 3B (30-44.9 mL/min)	12 (9.1%)	6 (4.3%)
Stage 4 (15-29.9 mL/min)	2 (1.5%)	3 (2.2%)
Baseline Albuminuria		
Normal (negative dipstick, ACR < 30 mg/g, or microalbumin < 30 mg/dL)	100 (75.8%)	97 (69.8%)
Mild (1+ dipstick, 30-300 mg/g ACR, or microalbumin > = 30 mg/dL)	24 (18.2%)	33 (23.7%)
Heavy (2+ dipstick, > 300 mg/g ACR)	8 (6.1%)	9 (6.5%)

There were *n* = 132 individuals randomized to REACH and *n* = 139 individuals assigned to control in the subsample

To understand how the intervention and baseline albuminuria impacted follow-up eGFR, we tested a linear regression with heteroscedasticity adjusted standard errors. Baseline eGFR significantly predicted follow-up eGFR (b = 0.846, SE = 0.035, *p* < .001), and there was a trending interaction effect of intervention and baseline albuminuria such that there may be a protective effect of REACH on eGFR among individuals with albuminuria (b = 6.016, SE = 3.440, *p* = .099). See Table 3 for full results.

**Table 3:** Linear regression predicting follow-up eGFR

	b	SE	t	*p*
Constant	12.16	.3115	3.890	<.001
Baseline eGFR	0.846	0.035	26.087	< 0.001
REACH Intervention	−1.180	1.787	−0.683	.50
Baseline Albuminuria	−3.327	2.353	−1.187	.24
Interaction	6.016	3.440	1.658	0.099

*eGFR* Estimated Glomerular filtration rate. REACH intervention = 1 and control = 0. Presence of albuminuria at baseline = 1 and absence = 0. Standard errors are heteroscedasticity adjusted

**Fig. 1:**
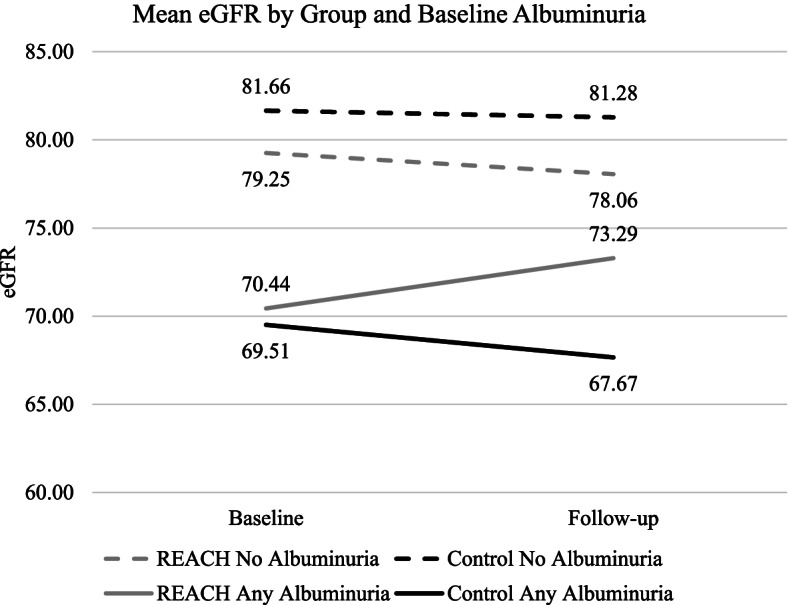
Estimated glomerular filtration rate (eGFR) at baseline and 12 months follow-up for REACH and Control separately by no and any albuminuria at baseline. Sample sizes for no albuminuria were N_Control_ = 97 and N_REACH_ = 100; sample sizes for any albuminuria were N_Control_ = 42 and N_REACH_ = 32

To better understand the possible interaction between baseline albuminuria and group assignment, we conducted non-parametric tests of difference (independent samples Mann-Whitney U-Tests) which are robust to non-normal distributions using a difference score in eGFR as the outcome. Difference scores for eGFR were computed such that a positive difference indicates improvement in eGFR while a negative difference indicates deterioration in eGFR from baseline to follow-up. For the no albuminuria group, median change in eGFR was 0.00 and − 1.43 for control and REACH, respectively. The distributions between REACH and control did not significantly differ for the no albuminuria group (*p* = .20, two-tailed). For the any albuminuria group, median change in eGFR was − 3.26 and 0.60 for control and REACH, respectively. There was no significant effect of REACH compared to control in change in eGFR from baseline to follow-up in the any albuminuria group (*p* = .11, two-tailed; see Fig. 1).

## Discussion

This study reports analyses from a patient sample representing individuals who are disproportionately burdened by kidney disease in the United States [30]. Further strengths of this work include intervention scalability as well as the inclusion of multimorbid individuals recruited from community health clinics who may be experiencing structural barriers to care. Within this diverse population of patients with T2D, 33.6% showed evidence of lower eGFR and/or albuminuria, which is slightly higher than other estimates [7, 12], emphasizing the high disease self-management needs of this group. Importantly, this study establishes the feasibility of conducting a behavioral, mobile health intervention for patients with T2D and comorbid kidney health concerns within a diverse sample [31].

Though exploratory analyses did not demonstrate that REACH resulted in a statistically significant improvement in eGFR at 12 months, the intervention may still have a clinically meaningful improvement in eGFR among those at higher risk for CKD progression. This could be due to several mechanisms. First, REACH improved HbA1c over 6 months [21], which via better diabetes control could have a positive impact on eGFR. Second, the REACH intervention improved adherence to diabetes medications [21], which may have also resulted in better medication adherence overall. Given that around 80% of the sample had hypertension, better adherence to hypertension medications could have a protective effect on eGFR. Finally, REACH may have changed the ways in which patients are thinking about their health or interacting with the healthcare system. Active engagement with clinicians and/or greater ownership of health goals and lifestyle improvements could serve to have additional positive effects on kidney function.

In the sub-sample with complete data, the REACH intervention did not demonstrate an association with change in eGFR over the 12-month study period in these exploratory analyses. Likewise in the parent trial, HbA1c reductions were no longer statistically significant at 12 months [21]. We followed-up a trending interaction effect between intervention and presence of albuminuria at baseline suggesting a possible protective effect of REACH on eGFR for individuals with albuminuria by investigating whether there was a stronger intervention effect for individuals with albuminuria at baseline to generate hypotheses for future work dedicated to this unanswered question. A recent large-scale effort, the Simultaneous Risk Factor Control Using Telehealth to slOw Progression of Diabetic Kidney Disease (STOP-DKD) study [32], found no main effects of the intervention on attenuation of kidney function decline. Secondary analyses reported significantly slower decline in eGFR by African American race; however, significant decline was observed for all races over 36 months [33]. Likewise, a mobile health intervention for individuals with T2D and hypertension was unsuccessful at lowering blood pressure over 12 months [34], highlighting the need for new approaches to mobile health intervention in this area.

Among those with no baseline albuminuria, there was relatively little change in eGFR over the 12-month study period for both the control and intervention groups. Importantly, there were no sudden deteriorations in eGFR as a result of participating in REACH. It is possible there may be long term benefits to tight glycemic control to prevent later development of CKD. Specifically, one study found 57% lower adjust risk for mild albuminuria and 84% lower risk of heavy albuminuria for patients with type 1 diabetes compared to those without treatment as well as differences in long term kidney functioning over 18 years [22]. However, these findings were over a significantly longer follow-up period, and therefore, effects from the REACH intervention may be evident at longer follow-up periods.

### Limitations

The context of the larger RCT contributed strengths and limitations to our exploratory analysis. First, we present secondary analyses and therefore are limited by the original study design and missing data. The resulting reduced sample size limited our power to detect differences between sub-groups. Second, many factors influence eGFR, all of which were not captured or accounted for in these analyses. Third, decline in eGFR is not a linear process [35], and our analyses did not allow for non-linear trajectories. Twelve months follow-up may be too short a time to see significant changes in eGFR, and a portion of those with diabetic kidney disease may initially have experienced hyperfiltration. However, the majority of values were collected between 9 and 13 months apart. Additionally, 71.8% of individuals in the subsample had an eGFR above 60 at baseline, indicating a lower risk for further rapid progression in the absence of adverse clinical events [36]. More robust differences between sub-groups may become apparent over longer follow-up. We did not evaluate for development of albuminuria or change in albuminuria over the study period, which would be important in future studies for a full description and understanding of kidney health in this population. Finally, additional data regarding other predictors and determinants of CKD progression, such as use of angiotensin converting enzyme inhibitors and sodium glucose co-transporter 2 inhibitors, fluctuations in blood pressure or intravascular volume, active tobacco use, and adherence to a diet low in animal protein were unavailable [37–39].

### Future directions

We aim to replicate analyses in a larger sample over a longer period of time to test the effects of a medication adherence intervention for T2D on CKD progression with an emphasis on individuals with albuminuria. The potentially promising results found here warrant future research efforts. Additionally, future research should investigate the mechanisms by which a medication adherence intervention positively impacts eGFR, including via higher adherence to all medications (not just diabetic medications), via biological changes due to medication effects (e.g. decreased HbA1c), or other mechanisms (e.g. emotional resilience, or other behavioral changes beyond medication adherence such as smoking cessation or increase in physical activity).

## Conclusions

The REACH intervention was successfully implemented in this vulnerable subpopulation with high comorbidity and risk for CKD progression. A concise, scalable, self-management intervention tailored to diabetes may have beneficial effects on kidney health, particularly among those at higher risk for CKD progression and those who are already experiencing structural barriers to care. Programs addressing diabetes management should align care to explicitly include and address kidney health. Further study is needed to determine if the intervention approach and content tested here are universally effective or may require adaptation for direct impact on CKD self-management and CKD progression.

## Data Availability

The datasets used and/or analyzed during the current study are available from the corresponding author on reasonable request.

## Abbreviations

T2D: Type 2 diabetes
CKD: Chronic kidney disease
SES: Socioeconomic status
eGFR: Estimated glomerular filtration rate
KDIGO: Kidney Disease Improving Global Outcomes
HbA1c: Hemoglobin A1c
REACH: Rapid Education/Encouragement and Communications for Health
RCT: Randomized control trial
EHR: Electronic health record
ICD: International Classification of Diseases
ACR: Albumin creatinine ratios
STOP-DKD: Simultaneous Risk Factor Control Using Telehealth to slOw Progression of Diabetic Kidney Disease
